# Oral Proteasomal Inhibitors Ixazomib, Oprozomib, and Delanzomib Upregulate the Function of Organic Anion Transporter 3 (OAT3): Implications in OAT3-Mediated Drug-Drug Interactions

**DOI:** 10.3390/pharmaceutics13030314

**Published:** 2021-02-28

**Authors:** Yunzhou Fan, Zhengxuan Liang, Jinghui Zhang, Guofeng You

**Affiliations:** Department of Pharmaceutics, Rutgers, The State University of New Jersey, 160 Frelinghuysen Road, Piscataway, NJ 08854, USA; yunzhou.fan@rutgers.edu (Y.F.); zhengxuan.liang@rutgers.edu (Z.L.); jinghui.zhang@rutgers.edu (J.Z.)

**Keywords:** drug transporter, ubiquitination, ixazomib, regulation

## Abstract

Organic anion transporter 3 (OAT3) is mainly expressed at the basolateral membrane of kidney proximal tubules, and is involved in the renal elimination of various kinds of important drugs, potentially affecting drug efficacy or toxicity. Our laboratory previously reported that ubiquitin modification of OAT3 triggers the endocytosis of OAT3 from the plasma membrane to intracellular endosomes, followed by degradation. Oral anticancer drugs ixazomib, oprozomib, and delanzomib, as proteasomal inhibitors, target the ubiquitin–proteasome system in clinics. Therefore, this study investigated the effects of ixazomib, oprozomib, and delanzomib on the expression and transport activity of OAT3 and elucidated the underlying mechanisms. We showed that all three drugs significantly increased the accumulation of ubiquitinated OAT3, which was consistent with decreased intracellular 20S proteasomal activity; stimulated OAT3-mediated transport of estrone sulfate and p-aminohippuric acid; and increased OAT3 surface expression. The enhanced transport activity and OAT3 expression following drug treatment resulted from an increase in maximum transport velocity of OAT3 without altering the substrate binding affinity, and from a decreased OAT3 degradation. Together, our study discovered a novel role of anticancer agents ixazomib, oprozomib, and delanzomib in upregulating OAT3 function, unveiled the proteasome as a promising target for OAT3 regulation, and provided implication of OAT3-mediated drug–drug interactions, which should be warned against during combination therapies with proteasome inhibitor drugs.

## 1. Introduction

Organic anion transporter 3 (OAT3), which is encoded by the SLC22A8 gene, is primarily expressed at the basolateral membrane of kidney proximal tubules, and actively translocates corresponding substrates from the blood into renal tubule epithelial cells. Those substrates are then effluxed out of the apical membrane into urine by other transporters [1,2,3]. OAT3 is involved in the renal elimination of various kinds of important clinical drugs from the kidney, such as anticancer agents (e.g., methotrexate), antivirals (e.g., tenofovir, valacyclovir), antibiotics (e.g., benzylpenicillin, cefotaxime), antihypertensives (e.g., furosemide, sitagliptin), H2 receptor antagonists (e.g., cimetidine, famotidine), and nonsteroidal anti-inflammatory drugs (e.g., ketoprofen, ibuprofen) [4,5,6]. Therefore, the renal OAT3 function is a critical determinant in drug clearance out of the body, and in the pharmacokinetic and pharmacodynamic properties of drugs, which ultimately affect the drugs’ efficacy and systemic or renal toxicity.

Combination therapies by coadministration of different drugs are often used for treatment of a single or multiple diseases. If one drug is an inhibitor, substrate, or inducer of OAT3, it will inhibit uncompetitively or competitively, or stimulate the renal transport and excretion of other clinical substrates, cause potential drug-drug interactions (DDIs), and sequent intra- and interindividual variation in clinical response to drugs [6,7]. Transporter-mediated clinical DDIs have attracted the attention of academic, industrial, and regulatory agencies. OAT3-mediated DDIs abundantly exist between imipenem-cilastatin, piperacillin-tazobactam, bezafibrate-mizoribine, puerarin-methotrexate, benzylpenicillin–acyclovir, etc., and markedly alter the pharmacokinetic parameters of affected drugs [8,9,10,11,12]. Through inhibition of OAT1/3, probenecid, wedelolactone, and wogonin prevented the kidney accumulation of aristolochic acid and related nephropathy, apigenin- or cilastatin-ameliorated imipenem, or diclofenac-induced nephrotoxicity [13,14,15,16,17].

The transporter expression and function may be modulated by certain drugs, phytomedicines, or xenobiotics, resulting in altered disposition of clinical substances, which is an indirect manner of obtaining transporter-mediated DDIs, in contrast to direct interaction with the transporter-like inhibitors or substrates [4]. For example, administration of 1α,25-dihydroxyvitamin D3, mercuric chloride, or methotrexate decreased OAT3 expression in crude or basolateral membranes of rat kidneys; while the renal expression was increased in normal rats by ochratoxin A treatment, in diabetic rats by insulin or atorvastatin plus insulin treatment, or in obese rats by prebiotic *Lactobacillus paracasei* HII01 or xylooligosaccharide treatment [18,19,20,21,22,23,24,25].

OAT3 expression and activity can be regulated through posttranslational modifications, including phosphorylation, ubiquitination, and SUMOylation [26,27,28]. As ubiquitination of OAT3 is an initiating process that triggers the internalization of OAT3 from the plasma membrane to intracellular endosomes, it is a critical molecular mechanism for OAT3 regulation [29,30]. Our lab demonstrated that activation of protein kinase C (PKC) could enhance OAT3 ubiquitination, and accelerate OAT3 internalization and subsequent degradation [27]. The transport activity and quantity of OAT3 on the plasma membrane were then reduced. Since proteasome inhibition can affect ubiquitination of targeted proteins and degradation, modulation of proteasome activity could potentially interfere with the physiological function of transporters. Proteasome inhibitors have shown to influence the copper transporter 1, Na^+^/H^+^ exchanger-3, ATP-binding cassette transporters A1 (ABCA1) and ABCG1, organic-anion-transporting polypeptide (OATP) 1B3, metal transporter ZIP14, and OAT1 [31,32,33,34,35,36]. However, it is not clear whether OAT3 can be regulated by controlling proteasome activity. Ixazomib, oprozomib, and delanzomib are oral proteasome inhibitors that target the ubiquitin–proteasome system for multiple myeloma treatment. In the present study, we investigated the influence of ixazomib, oprozomib, and delanzomib on OAT3 expression and transport activity, and elucidated the underlying mechanisms.

## 2. Materials and Methods

### 2.1. Materials

COS-7 cells and HEK293 cells were purchased from ATCC (Manassas, VA, USA). [^3^H]-labeled estrone sulfate (ES) and [^3^H]-labeled p-aminohippuric acid (PAH) were ordered from PerkinElmer (Waltham, MA, USA). Mouse anti-Myc antibody (9E10) was purchased from Roche (Indianapolis, IN, USA). Mouse anti-E-Cadherin antibody was from Abcam (Cambridge, MA, USA). Streptavidin agarose resin, protein G agarose, and Sulfo-NHS-SS-biotin were ordered from Thermo Scientific (Rockford, IL, USA). The 20S proteasome assay kit was ordered from Cayman Chemical Company (Ann Arbor, MI, USA). Mouse anti-β-actin antibody, normal mouse IgG, and mouse anti-ubiquitin antibody were obtained from Santa Cruz Biotechnology (Dallas, TX, USA). Ixazomib, oprozomib, and delanzomib were purchased from Selleck Chemicals (Houston, TX, USA). Probenecid, lactacystin, epoxomicin and all other reagents were purchased from Sigma-Aldrich (St. Louis, MO, USA).

### 2.2. Cell Culture

Parental COS-7 and parental HEK293 cells were cultured in Dulbecco’s modified Eagle’s medium (DMEM) (Corning, Tewksbury, MA, USA) supplemented with 10% fetal bovine serum (Gibco, Grand Island, NY, USA) at 37 °C in 5% CO_2_. Human OAT3-expressing (hOAT3) COS-7 cells and hOAT3-expressing HEK293 cells were established in our group [37,38]. The hOAT3 cells were cultured in DMEM supplemented with 10% fetal bovine serum and 0.2 mg/mL G418 sulfate (Gibco, Grand Island, NY, USA).

### 2.3. Transport Measurement

The transport activity was assayed using the method published by our lab [30]. Cells per well were incubated in uptake solution of [^3^H]ES (250 nM) or [^3^H]PAH (20 µM) in phosphate-buffered saline (PBS)/Ca^2+^/Mg^2+^ (PBS/CM) for 3 min. After discarding the uptake solution, the cells were washed twice with cold PBS, then lysed in NaOH solution (0.2 N) and neutralized by adding HCl solution (0.2 N). The amount of ES or PAH uptake was assayed using a Beckman LS 6500 liquid scintillation counter.

### 2.4. 20S Proteasome Activity Assay

After incubation with ixazomib, oprozomib, delanzomib, or lactacystin for 6 h, hOAT3 cells were washed once with assay buffer (200 µL) and solubilized in lysis buffer (100 µL). Then, the supernatant (90 µL) was removed to a black 96-well plate, and incubated with SUC-LLVY-AMC solution (10 µL) for 1 h at 37 °C. Fluorescence intensity per well (excitation = 360 nm, emission = 480 nm) was assayed using a Molecular Devices Spectramax M3 microplate reader.

### 2.5. Cell-Surface Biotinylation

Cell surface hOAT3 expression was assayed using the procedures introduced by our group [39]. The hOAT3 cells were labeled with sulfo-NHS-SS-biotin solution (0.5 mg/mL in PBS/CM) on ice, with slow shaking for two continuous 20 min. After discarding the biotin solution, the cells were washed once with glycine solution (100 mM in PBS/CM) and incubated with glycine solution for 20 min to completely quench the unbound sulfo-NHS-SS-biotin. The cells were then lysed in lysis buffer consisted of 10 mM Tris-HCl, pH 7.5, 150 mM NaCl, 1 mM EDTA, 0.1% SDS, 1% Triton X-100, and 1% proteinase inhibitor cocktail. The cell lysates were centrifuged at 16,000× *g* at 4 °C, and the supernatant was then mixed with streptavidin agarose resin (40 µL) to separate the cell surface proteins. The hOAT3 at the cell surface was detected by immunoblotting using the anti-Myc antibody.

### 2.6. Immunoprecipitation

The hOAT3 ubiquitination was investigated using the method published by our group [39]. The hOAT3 cells were lysed in lysis buffer consisted of 50 mM Tris-HCl, pH 8.0, 150 mM NaCl, 1% Triton X-100, 10% glycerol, 5 mM EDTA, 1 mM NaF, 20 mM N-ethylmaleimide, and 1% of proteinase inhibitor cocktail. Cell lysates were precleared with protein G agarose to decrease nonspecific binding at 4 °C for 2 h. Anti-Myc antibody was mixed with protein G agarose (30 µL) and incubated at 4 °C for 2 h. The precleared protein was then added to antibody-bound protein G agarose suspension and mixed with end-over-end rotation at 4 °C overnight. Proteins coupled to protein G agarose were released with urea buffer containing β-mecaptoethanol and detected by immunoblotting using the anti-ubiquitin antibody.

### 2.7. Degradation Assay of OAT3

The hOAT3 degradation was investigated using the method utilized in our group [38]. The hOAT3 cells were first labeled with sulfo-NHS-SS-biotin, then the biotinylated cells were treated with vehicle, ixazomib, oprozomib, or delanzomib at 37 °C for 0, 3, and 6 h. Then the cells were collected, and the undegraded cell surface hOAT3 was isolated and detected following the procedures in Section 2.5.

### 2.8. Electrophoresis and Immunoblotting

The electrophoresis and immunoblotting experiments were carried out using the method published by our group [30]. Protein samples were loaded on 7.5% precast polyacrylamide gels and transferred onto polyvinylidene difluoride membranes. The immunoblot membranes were blocked with 5% nonfat dry milk in PBS-0.05% tween 20 for 1 h, and incubated with primary antibodies at 4 °C overnight, followed by incubation of horseradish peroxidase-conjugated secondary antibodies. The protein bands were visualized using a SuperSignal West Dura Extended Duration Substrate kit (Thermo Scientific, Rockford, IL, USA), and corresponding densities were analyzed using the FluorChem 8000 imaging system (Alpha Innotech Corp., San Leandro, CA, USA).

### 2.9. Data Analysis

One-way ANOVA or two-way ANOVA Tukey’s test was utilized for statistical analysis among multiple groups by using GraphPad Prism software (GraphPad Software Inc., San Diego, CA, USA). Each experiment was repeated at least three times. A *p* value less than 0.05 was statistically significant, and a *p* value more than 0.05 was not statistically significant (ns).

## 3. Results

### 3.1. Effects of Ixazomib, Oprozomib, and Delanzomib on the Ubiquitination of OAT3

**Figure 1:**
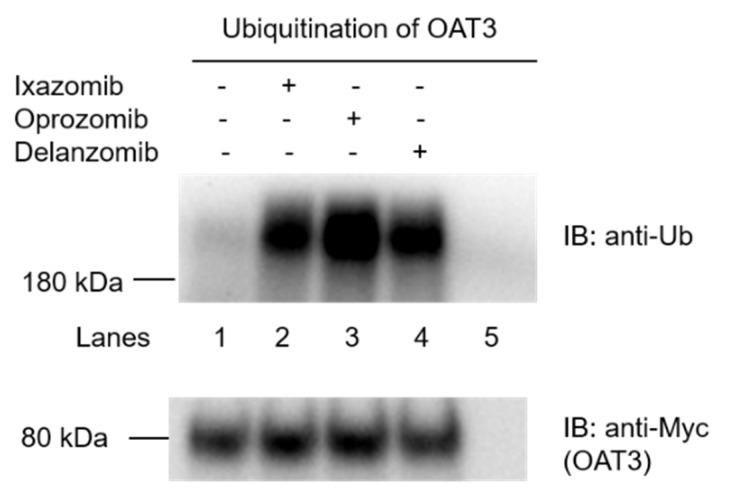
Effect of proteasomal inhibitors ixazomib, oprozomib, or delanzomib on the accumulation of ubiquitinated OAT3. Top panel: OAT3-expressing COS-7 cells were treated with ixazomib (30 nM), oprozomib (200 nM), or delanzomib (30 nM) for 6 h. Treated cells were then lysed, and OAT3 was immunoprecipitated with anti-Myc antibody or mouse IgG (as negative control, lane 5), followed by IB with anti-Ub. Bottom panel: The same immunoblot from the top panel was reprobed with anti-Myc antibody to determine the amount of OAT3 immunoprecipitated.

**Figure 2:**
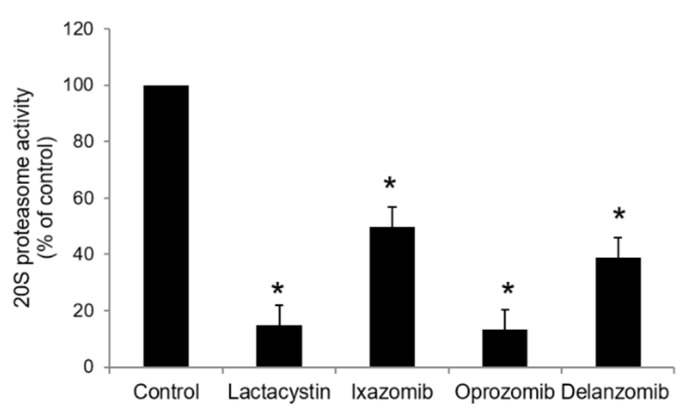
Effect of ixazomib, oprozomib, or delanzomib on the 20S proteasome activity. OAT3-expressing COS-7 cells were treated with lactacystin (10 μM), a classical proteasome inhibitor as positive control, ixazomib (30 nM), oprozomib (200 nM), or delanzomib (30 nM) for 6 h. The 20S proteasome activity of cells was then performed. The 20S proteasome activity was expressed as the percentage of control cells from three independent experiments. Values are means ± S.D. (n = 3). * *p* < 0.05.

Ixazomib, oprozomib, and delanzomib, as proteasome inhibitors, target the ubiquitin-proteasome system for cancer therapy. First, we investigated their effects on the intracellular ubiquitination of OAT3 in OAT3-expressing COS-7 cells. OAT3-expressing cells were treated with ixazomib, oprozomib, or delanzomib for 6 h, then harvested and lysed. OAT3 was pulled down from cell lysate by anti-Myc antibody (Myc tag was fused onto OAT3, enabling immunodetection), followed by immunoblotting (IB) using anti-ubiquitin antibody to probe the ubiquitinated OAT3. The results (Figure 1) showed that incubating cells with ixazomib, oprozomib, or delanzomib resulted in a significant accumulation of ubiquitinated OAT3, which was not because of the difference in immunoprecipitated OAT3, since there were similar quantities of OAT3 pulled down from all samples. Further study showed that like lactacystin, a classical proteasome inhibitor, ixazomib, oprozomib, and delanzomib inhibited the 20S proteasome activity by 50% (95% confidence interval (CI): 46% to 54%), 87% (95% CI: 83% to 91%), and 61% (95% CI: 57% to 65%), respectively, after 6 h of treatment (Figure 2). Therefore, the accumulation of ubiquitinated OAT3 was attributed to the decreased proteasome activity, suggesting that ubiquitinated OAT3 can be modulated by interfering the ubiquitin–proteasome system in the cell model that we used.

### 3.2. Cis-Effect of Ixazomib, Oprozomib, or Delanzomib on OAT3-Mediated Uptake of Estrone Sulfate

**Figure 3:**
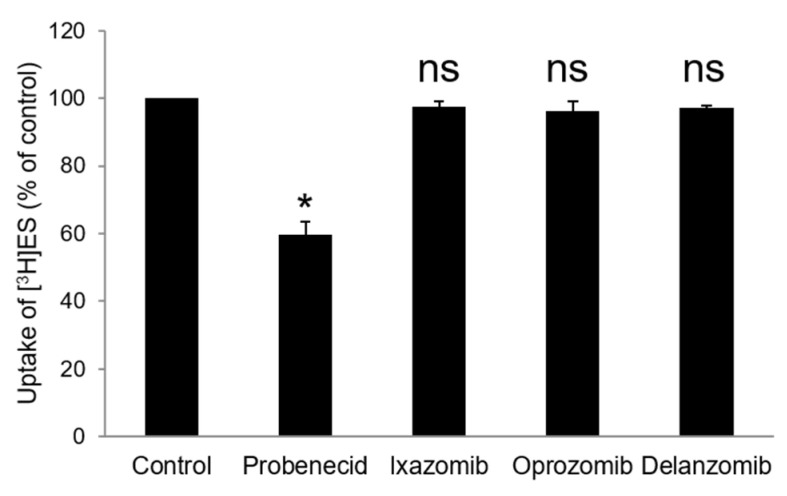
Cis-effect of ixazomib, oprozomib, or delanzomib on OAT3-mediated uptake of [^3^H]ES. The uptake of [^3^H]ES (250 nM) in the presence of ixazomib (1 μM), oprozomib (1 μM), delanzomib (1 μM), or probenecid (5 μM) for 3 min was measured in OAT3-expressing COS-7 cells. Each data point represented only carrier-mediated transport after subtraction of values from parental cells. Uptake activity was expressed as the percentage of uptake measured in control cells from three independent experiments. Values are means ± S.D. (n = 3). * *p* < 0.05; ns = not statistically significant.

As OAT3 has multispecificity toward multiple substrates, we investigated whether ixazomib, oprozomib, and delanzomib are inhibitors or inducers of OAT3 by performing a cis-inhibition assay. Estrone sulfate (ES) is a prototypical OAT3 substrate, and probenecid is a well-recognized competitive inhibitor of OAT3 [2,40]. We measured 3 min of uptake of [^3^H]ES (250 nM) into OAT3-expressing cells with or without probenecid, ixazomib, oprozomib, or delanzomib existing in the ES solution. The results (Figure 3) showed that probenecid inhibited OAT3-mediated transport of [^3^H]ES by 40% (95% CI: 34% to 46%), while ixazomib, oprozomib, and delanzomib did not have any effects, indicating that ixazomib, oprozomib, and delanzomib are not inhibitors or inducers of OAT3. Therefore, ixazomib, oprozomib, and delanzomib did not affect OAT3 function through direct interaction with the transporter.

### 3.3. Effects of Ixazomib, Oprozomib, or Delanzomib on OAT3-Mediated Uptake of Estrone Sulfate or P-Aminohippuric Acid

**Figure 4:**
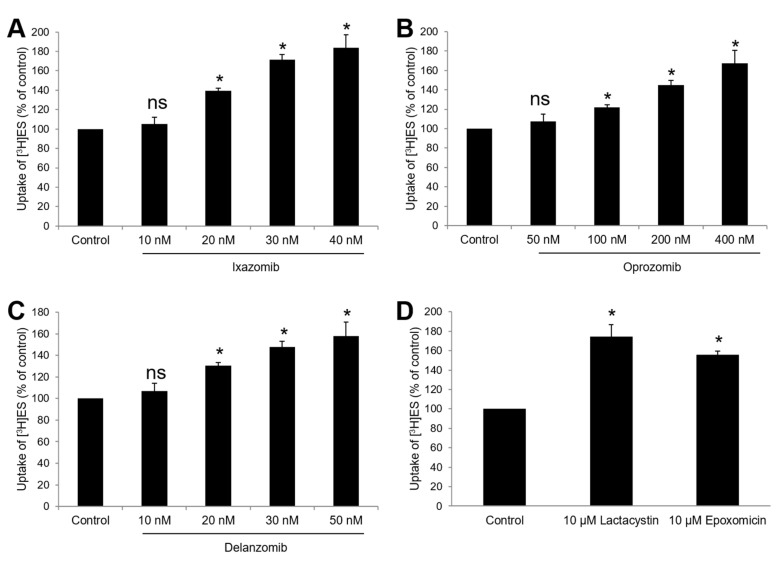
Effect of ixazomib, oprozomib, delanzomib, or classical proteasome inhibitors on OAT3 activity. OAT3-expressing COS-7 cells were treated with ixazomib (**A**), oprozomib (**B**), delanzomib (**C**), or classical proteasome inhibitors lactacystin or epoxomicin (**D**) at indicated concentrations for 6 h. The uptake of [^3^H]ES (250 nM) for 3 minu was then performed. Each data point represented only carrier-mediated transport after subtraction of values from parental cells. Uptake activity was expressed as the percentage of uptake measured in control cells from three independent experiments. Values are means ± S.D. (n = 3). * *p* < 0.05; ns = not statistically significant.

**Figure 5:**
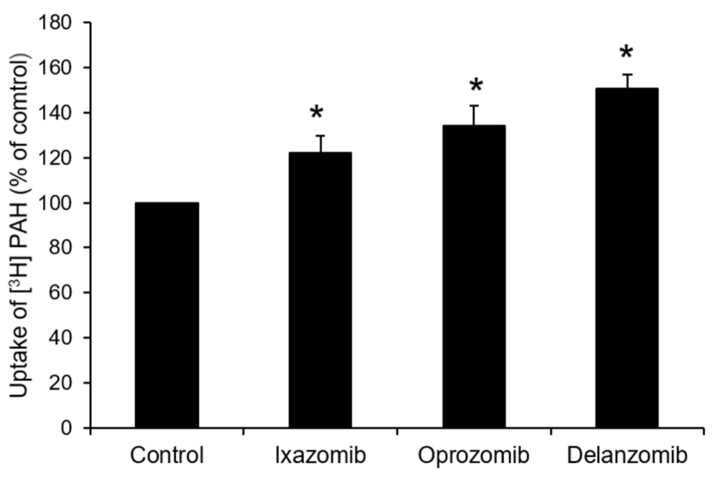
Effect of ixazomib, oprozomib, or delanzomib on OAT3-mediated transport of p-aminohippuric acid. OAT3-expressing COS-7 cells were treated with ixazomib (30 nM), oprozomib (200 nM), or delanzomib (30 nM) for 6 h. The uptake of [^3^H]PAH (20 µM) for 3 min was then performed. Each data point represented only carrier-mediated transport after subtraction of values from parental cells. Uptake activity was expressed as the percentage of uptake measured in control cells from three independent experiments. Values are means ± S.D. (n = 3). * *p* < 0.05.

**Figure 6:**
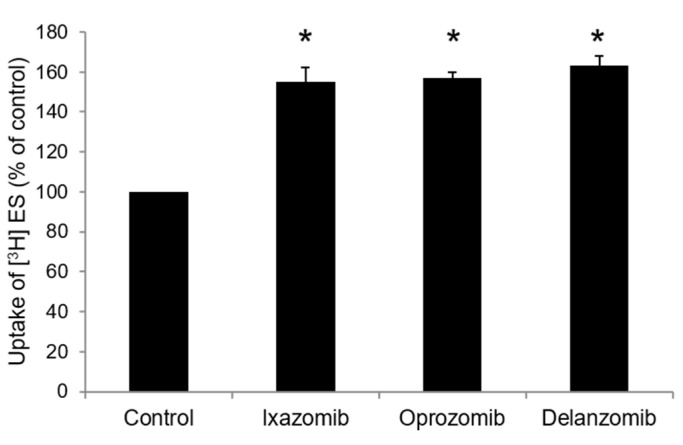
Effect of ixazomib, oprozomib, or delanzomib on OAT3 activity in OAT3-expressing HEK293 cells. OAT3-expressing HEK293 cells were treated with ixazomib (30 nM), oprozomib (200 nM), or delanzomib (30 nM) for 6 h. The uptake of [^3^H]ES (250 nM) for 3 min was then performed. Each data point represented only carrier-mediated transport after subtraction of values from parental cells. Uptake activity was expressed as the percentage of uptake measured in control cells from three independent experiments. Values are means ± S.D. (n = 3). * *p* < 0.05.

**Figure 7:**
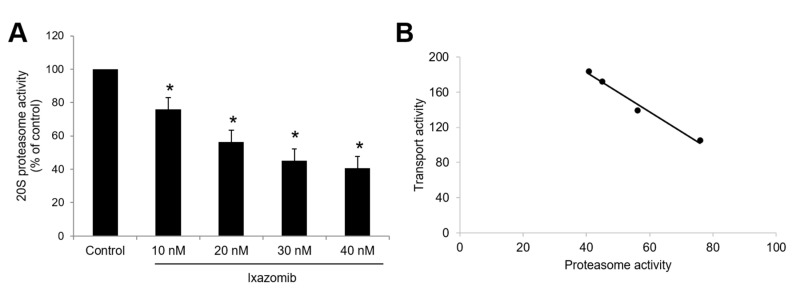
Dose-effect of ixazomib on the 20S proteasome activity. (**A**) OAT3-expressing COS-7 cells were treated with ixazomib at indicated concentrations for 6 h. The 20S proteasome activity of cells was then performed. The 20S proteasome activity was expressed as the percentage of control cells from three independent experiments. Values are means ± S.D. (n = 3). * *p* < 0.05. (**B**) Correlation analysis was performed between transport activity from Figure 4A and proteasomal activity from Figure 7A after ixazomib treatment.

Since ixazomib, oprozomib, and delanzomib can increase OAT3 ubiquitination, we further investigated their effect on the transport activity. OAT3-expressing cells were treated with ixazomib, oprozomib, or delanzomib for 6 h, then OAT3-mediated uptake of ES was measured. The results (Figure 4A–C) showed that ixazomib, oprozomib, and delanzomib all induced a dose-dependent stimulation of ES uptake in OAT3-expressing COS-7 cells. The OAT3 transport activity was stimulated by 72% (95% CI: 46% to 97%), 45% (95% CI: 33% to 56%), and 48% (95% CI: 31% to 64%) at 30 nM ixazomib, 200 nM oprozomib, and 30 nM delanzomib, respectively. Consistently, 6 h of treatment with classical proteasome inhibitors lactacystin or epoxomicin stimulated the uptake of ES (Figure 4D). Besides, p-aminohippuric acid (PAH) is another OAT3 substrate [41,42]. Like ES, our result (Figure 5) showed that all three proteasome inhibitor drugs also significantly stimulated PAH uptake in a substrate-independent manner. Similar stimulative effects also existed in OAT3-expressing HEK293 cells, excluding cell-specific effects of proteasome inhibitors (Figure 6). Further study showed that 10~40 nM ixazomib induced a dose-dependent inhibition of proteasome activity (Figure 7A), and there was a strongly association between transport activity and proteasomal activity (correlation coefficient was 0.98, Figure 7B). We selected the concentration of 30 nM ixazomib, 200 nM oprozomib, and 30 nM delanzomib for the following mechanisms study.

### 3.4. Kinetic Analysis of the Effects of Ixazomib, Oprozomib, or Delanzomib on OAT3-Mediated Uptake of Estrone Sulfate

**Figure 8:**
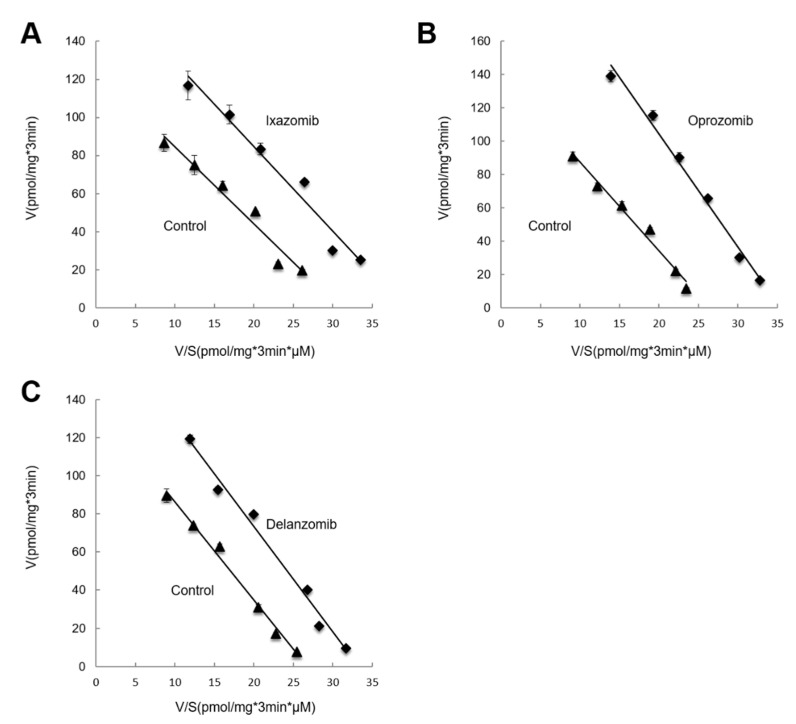
Effect of ixazomib, oprozomib, or delanzomib on the kinetics of hOAT3-mediated estrone sulfate transport. OAT3-expressing COS-7 cells were treated with 30 nM ixazomib (**A**), 200 nM oprozomib (**B**), or 30 nM delanzomib (**C**) for 6 h, and initial uptake (3 min) of [^3^H]ES was measured at the concentration of 0.3~10 µM. The data represent uptake into hOAT3-expressing cells minus uptake into mock cells (parental COS-7 cells). Values are means ± S.D. (n = 3). V = velocity; S = substrate concentration.

To examine the mechanism of ixazomib-, oprozomib-, and delanzomib-induced stimulation of OAT3 activity, we determined [^3^H]ES uptake at a series of concentrations (0.3~10 µM). Eadie–Hofstee analyses of the derived data (Figure 8) showed that incubation of ixazomib (Figure 8A), oprozomib (Figure 8B), or delanzomib (Figure 8C) resulted in an increased maximum transport velocity V_max_ (128 ± 3 pmol·mg^−1^·3 min^−1^ with untreated cells and 176 ± 7 pmol·mg^−1^·3 min^−1^ in the presence of ixazomib; 130 ± 2 pmol·mg^−1^·3 min^−1^ with untreated cells and 223 ± 4 pmol·mg^−1^·3 min^−1^ in the presence of oprozomib; 128 ± 11 pmol·mg^−1^·3 min^−1^ with untreated cells and 175 ± 11 pmol·mg^−1^·3 min^−1^ in the presence of delanzomib), with no significant change of substrate-binding-affinity Km for ES (4.2 ± 0.3 μM with untreated cells and 4.6 ± 0.4 μM in the presence of ixazomib; 4.6 ± 0.1 μM with untreated cells and 6.0 ± 0.2 μM in the presence of oprozomib; 4.3 ± 0.9 μM with untreated cells and 4.9 ± 0.7 μM in the presence of delanzomib). These results indicated that stimulated activity of ixazomib, oprozomib, and delanzomib resulted from an increase of the transport rate of OAT3, and not from an enhanced affinity at the substrate-binding site.

### 3.5. Effect of Ixazomib, Oprozomib, or Delanzomib on OAT3 Expression

**Figure 9:**
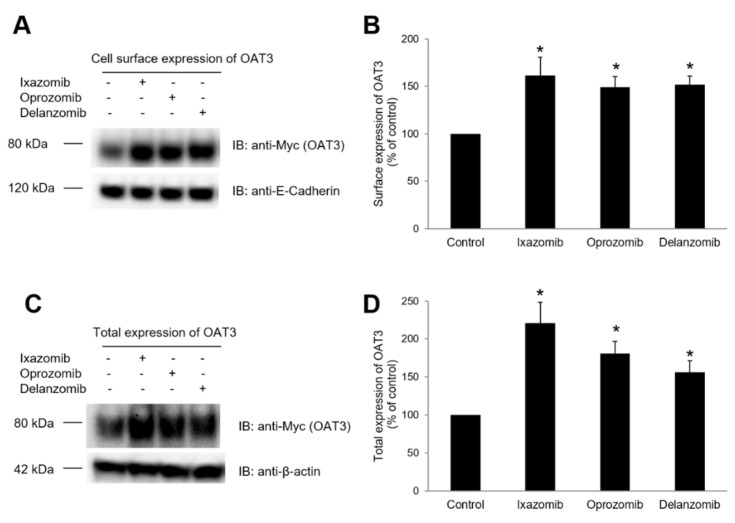
Effect of ixazomib, oprozomib, or delanzomib on OAT3 expression. (**A**) Top panel: OAT3-expressing COS-7 cells were treated with ixazomib (30 nM), oprozomib (200 nM), or delanzomib (30 nM) for 6 h. Cell-surface biotinylation was performed. Biotinylated (cell surface) proteins were separated with using streptavidin agarose resin and analyzed by IB with an anti-Myc antibody. Bottom panel: The same blot from the top panel was reprobed with an anti-E-Cadherin antibody. E-Cadherin is an integral membrane protein marker. (**B**) Densitometry plot of results from (**A**), top panel, as well as from other experiments. Values are means ± S.D. (n = 3). * *p* < 0.05. (**C**) Top panel: OAT3-expressing COS-7 cells were treated with ixazomib (30 nM), oprozomib (200 nM), or delanzomib (30 nM) for 6 h. Cells were then lysed, followed by IB with anti-Myc antibody. Bottom panel: The same blot from the top panel was reprobed with an anti-β-actin antibody. β-actin is a cellular protein marker. (**D**) Densitometry plot of results from (**C**), top panel, as well as from other experiments. Values are means ± S.D. (n = 3). * *p* < 0.05.

As ixazomib, oprozomib, and delanzomib did not alter the binding affinity of OAT3, the increase of transport activity may mainly result from the increased expression on the plasma membrane. OAT3-expressing cells were treated with ixazomib, oprozomib, or delanzomib for 6 h, and OAT3 expression on the plasma membrane and in the whole cell lysates were investigated. The result showed that treatment with ixazomib, oprozomib, or delanzomib all caused an increase of OAT3 expression on the cell membrane (Figure 9A,B) and in the whole cell lysate (Figure 9C,D), which was not because of the overall interference in cellular proteins, as there were similar quantities of membrane fraction marker E-Cadherin (Figure 9A) and whole cellular fraction marker β-actin (Figure 9C) in all samples.

### 3.6. Effect of Ixazomib, Oprozomib, and Delanzomib on OAT3 Degradation

**Figure 10:**
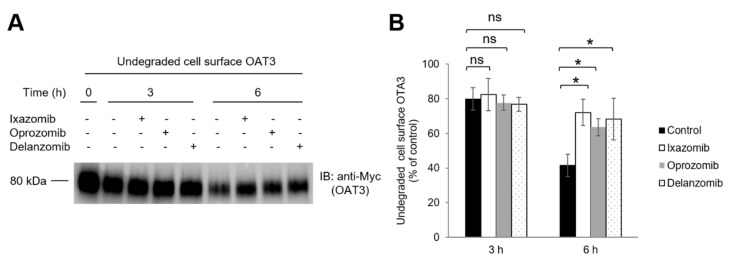
Effect of ixazomib, oprozomib, or delanzomib on OAT3 stability. (**A**) OAT3-expressing COS-7 cells were biotinylated with membrane-impermeable biotinylation reagent sulfo-NHS-SS-biotin. Labeled cells were then treated with ixazomib (30 nM), oprozomib (200 nM), or delanzomib (30 nM) at 37 °C for 3 and 6 h, respectively. Treated cells were lysed, and cell-surface proteins were isolated using streptavidin-agarose resin, followed by IB with anti-Myc antibody. (**B**) Densitometry plot of results from (**A**), as well as from other experiments. The amount of undegraded cell-surface hOAT3 was expressed as % of total initial cell-surface hOAT3 pool. Values are means ± S.D. (n = 3). * *p* < 0.05; ns = not statistically significant. Two-way ANOVA Tukey’s test was applied for statistical analysis.

The ubiquitin–proteasome pathway ultimately affects the degradation of targeted proteins, therefore the degradation of cell-membrane OAT3 was investigated by biotinylation and isolation of cell-surface proteins. OAT3-expressing cells were first labeled with sulfo-NHS-SS-biotin on all membrane proteins at 4 °C, then biotinylated cells were incubated with ixazomib, oprozomib, or delanzomib for 3 and 6 h at 37 °C. At the time points, those cells were harvested and lysed, and cell-membrane proteins were enriched in streptavidin agarose beads, followed by immunoblotting detection of OAT3 using anti-Myc antibody. The results (Figure 10) revealed that compared to control, the degradation of cell membrane OAT3 was reduced markedly after 6 h incubation of the three drugs, and without effect at 3 h, indicating that ixazomib, oprozomib, and delanzomib chronically enhanced the stability of membrane OAT3.

## 4. Discussion

OAT3 function is predominantly dependent on the amount located on the plasma membrane, which can be regulated by mitogen-activated protein kinase (MAPK), protein kinase A (PKA), PKC signaling pathways [43,44,45]. Ubiquitination is a significant posttranslational mechanism for OAT3 regulation. Our previous study had demonstrated the essential role of Nedd4-2 (a ubiquitin ligase) in the ubiquitination, surface expression, and transport activity of OAT3 [27]. Serum- and glucocorticoid-inducible kinases 1 (sgk1), PKC, janus tyrosine kinase 2 (JAK2) regulated OAT3 through Nedd4-2, which showed Nedd4-2 is molecular target for OAT3 regulation [27,37,38,46]. In this study, we further discovered proteasome was a novel target for regulation of OAT3 and stimulating OAT3 function can be achieved through inhibiting proteasomal activity.

COS-7 and HEK293 cells lacking in endogenous OATs were commonly utilized as heterologous expression systems for OATs. Both cell lines were broadly selected for study the regulation and mechanisms of the cloned OATs and other drug transporters in kidney with several advantages [13,47,48,49]. Expression of exogenous OAT3 will allow us to study the transport characteristics of OAT3 without being disturbed by other OATs. They are originated from the kidney and have the proteasome activity and signaling pathways involved in OAT3 regulation. COS-7 cells and HEK293 cells used in our studies will provide the research basis for the upcoming work focusing on validating whether primary epithelia possess the similar mechanisms.

Ixazomib is an FDA-approved anticancer drug, while oprozomib and delanzomib are in phases of clinical trials. All of them are administered orally, and preferentially bind reversibly (ixazomib and delanzomib) or irreversibly (oprozomib) and inhibit the chymotrypsin-like activity of the 20S proteasome in various tissues and organs. There were reports that ixazomib inhibited the proteasome activity in the whole blood and tumor; oprozomib could inhibit the proteasome activity in the blood, peripheral blood mononuclear cells, liver, kidney, and adrenal glands; and delanzomib inhibited the proteasome activity in blood mononuclear cells, kidney, and spleen [50,51,52,53,54,55]. Ixazomib prevented antibody-mediated rejection in kidney transplantation and treated patients with metastatic kidney cancer [56,57]. Delanzomib can ameliorate lupus nephritis in mice [55]. These results suggested that proteasomal inhibitors can be used to treat kidney diseases, through proteasome inhibition-mediated reduction in aberrant cytokines and antibodies, or downregulation of nuclear factor kappa B-dependent gene expression and resulted tumor growth [58,59].

Ixazomib, oprozomib, or delanzomib treatment substantially increased the accumulation of ubiquitinated OAT3 (Figure 1), which was consistent with decreased 20S proteasomal activity in cell lysate in OAT3-expressing cells (Figure 2), stimulated OAT3-mediated transport of estrone sulfate and p-aminohippuric acid (Figure 4, Figure 5 and Figure 6), and increased OAT3 membrane expression (Figure 9). The enhanced transport activity of OAT3 following drug pretreatment resulted from an increase in maximum transport velocity without altering the binding affinity of the transporter (Figure 8). Ubiquitinated OAT3 exhibited the molecular mass above 180 kDa, ~100 kDa more than OAT3 (~80 kDa). As ubiquitin is an 8-kDa polypeptide, OAT3 may be modified by poly- or multiubiquitination (Figure 1).

The OAT3 function was chronically stimulated with 6 h of treatment with ixazomib, oprozomib, or delanzomib. As the alteration of trafficking processes, including internalization or recycling of OATs, can be reflected in function change during acute regulation (such as 0.5 h), we can exclude the reduced internalization and increased recycling that are the underlying mechanisms for those drugs [27,28,29,30,39]. With further exploring, the degradation of OAT3 was decelerated by ixazomib, oprozomib, or delanzomib (Figure 10). Our results showed they inhibited the 20S proteasome activity (Figure 2), and there was a negative association between proteasomal activity and transport activity at 10–40 nM ixazomib (Figure 7B). Together, ixazomib-, oprozomib-, and delanzomib-upregulated OAT3 function was mainly through suppression of proteasome activity and decelerated degradation of OAT3.

The concentrations of ixazomib (10–40 nM), oprozomib (50–400 nM), and delanzomib (10–50 nM) used in our study are in the clinically therapeutic range. After once-weekly oral dosing of 2.23 mg/m^2^ for 3 weeks in combination therapy with lenalidomide and dexamethasone, the mean maximum plasma concentration (C_max_) of ixazomib in multiple myeloma patients at day 1 and day 15 was 22.3 ng/mL (61.7 nM) and 31.4 ng/mL (87.0 nM), respectively [60]. For oprozomib, after 2 consecutive days weekly oral dosing at 210 mg/day for 4 weeks plus pomalidomide and dexamethasone in relapsed/refractory multiple myeloma patients, the mean C_max_ of oprozomib at day 1 and day 8 was 744 ng/mL (1.4 µM) and 1030 ng/mL (1.9 µM), respectively [61]. Until now, there were only reports about intravenous pharmacokinetic data of delanzomib in human. After 2 days weekly intravenous dosing 0.4–1.8 mg/m^2^ for 2 weeks in patients with solid tumors and multiple myeloma, the mean C_max_ of delanzomib on day 1 was 88.4–557.3 ng/mL (0.2–1.3 µM) [54]. Ixazomib and delanzomib have a long terminal plasma half-life of 3.6–11.3 days and 62.0 ± 43.5 h, respectively [54,62]. Though oprozomib has a short plasma half-life of about 1 h resulting from rapid systemic clearance, the recovery of proteasome activity in tissues needed a longer time of 24~72 h due to irreversible binding [63,64]. Therefore, the inductive effects of ixazomib, oprozomib, and delanzomib on drug elimination and DDIs potentially exist, though they are administered once or twice weekly. The in vitro regulation and related mechanisms in cell models were reported in this study, and further in vivo study in Sprague Dawley rats by oral ixazomib will be performed to further explore the roles of ixazomib in proteasome activity, OTA3 ubiquitination, drug uptake in kidney slices, membrane and total expression in kidney, and renal clearance of drugs by kidney in our lab.

Ixazomib, oprozomib, or delanzomib are all indicated in combination with dexamethasone, a synthetic glucocorticoid for the treatment of patients with multiple myeloma [61,65,66]. Our previous study showed dexamethasone stimulates OAT3 transport activity and membrane expression through the serum- and glucocorticoid-inducible kinases 1 signaling pathway, suggesting the stimulatory effect on OAT3 may be further magnified using ixazomib, oprozomib, and delanzomib in combination with dexamethasone [37].

Ixazomib is a low-affinity substrate of P-glycoprotein (P-gp); is not a substrate of breast cancer resistance protein (BCRP), multidrug resistance protein 2 (MRP2), or hepatic OATPs; and is not an inhibitor of P-gp, BCRP, MRP2, OATP1B1, OATP1B3, organic cation transporter 2 (OCT2), OAT1, OAT3, multidrug and toxin extruder 1 (MATE1), or MATE2-K. Therefore, the manufacturer claimed that ixazomib is not expected to cause transporter-mediated drug–drug interactions [67]. Consistent with this, our study found that ixazomib is not an inhibitor of OAT3 (Figure 3). However, although ixazomib did not cause DDIs through direct interaction (inhibiting or competing) with the transporters, our study showed that ixazomib can upregulate OAT3 activity through induced membrane expression, which may affect the disposition of other drugs in an indirect manner of transporter-mediated DDIs. Besides, potential DDIs may be occurred by direct OATs induction. There were reports that ursolic acid and ciprofloxacin stimulated OAT1-mediated p-aminohippuric acid uptake, and 1,5-dicaffeoylquinic acid and 18β-glycyrrhetinic acid stimulated hOAT4-mediated estrone sulfate uptake [68,69].

Proteasome inhibition drugs are now well utilized for cancer treatment. In contrast, impaired proteasome function and related elevation of toxic intracellular protein or aggregates are involved in neurodegenerative disorders (e.g., Parkinson’s disease, Alzheimer’s disease) and cardiac dysfunctions, and enhancement of proteasome activity may also be a promising therapeutic strategy for those diseases [70,71,72]. PD169316, pyrazolones and chlorpromazine as small molecules, were found to be proteasome activators [70,73,74]. It would be interesting to study whether proteasomal activators can regulate the OAT3 function.

Our findings that oral proteasome inhibitors ixazomib, oprozomib, and delanzomib can increase OAT3 transport activity have important physiological implications. First, it can accelerate the drugs clearance from body, resulting in reduced plasma concentration and therapeutic efficacy of drugs. We can also use this mechanism for noninvasive detoxification in the event of drug overdoses. Second, it may enhance the entering and distribution of drugs in proximal tubular cells, leading to potential nephrotoxicity. Those points should attract the attention of physicians and pharmacists for rational use of medicines and irrational drug combinations, and avoiding potential drug–drug interactions. Third, bilateral ureteral obstruction (BUO), a common clinical disease, impaired renal elimination of drugs partly resulted from reduced cell-surface expression of OAT3 [75]. Proteasome inhibition may provide a potential strategy to reverse BUO or other kidney-disease-induced downregulation of OAT3. Last, it also can promote renal clearance of toxins, metabolites, signaling molecules, nutrients, and other substances as OAT3 substrates, and maintain homeostasis within the body.

## 5. Conclusions

**Figure 11:**
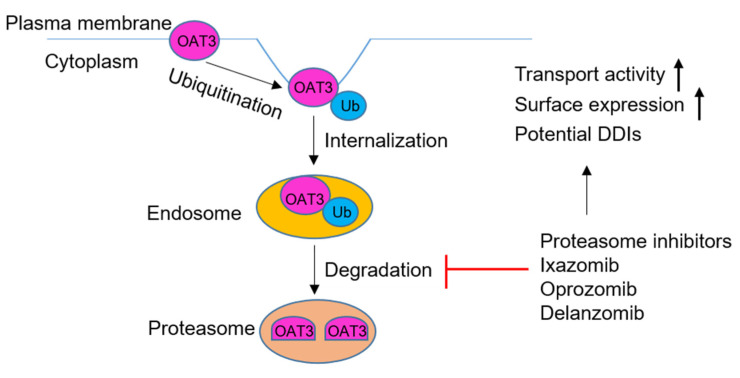
Oral proteasomal inhibitors ixazomib, oprozomib, and delanzomib upregulate the transport activity and expression of OAT3. Ub = ubiquitin; DDIs = drug–drug interactions.

Our studies showed for the first time that anticancer drugs ixazomib, oprozomib, and delanzomib had a critical role in upregulating OAT3 transport activity and expression, suggesting their potential impact on the OAT3-mediated drug disposition and clinical drug–drug interactions during combination therapies of proteasome inhibitor drugs and other types of drugs (Figure 11).

## Data Availability

Not applicable.
